# Altered Outer Membrane Transcriptome Balance with AmpC Overexpression in Carbapenem-Resistant *Enterobacter cloacae*

**DOI:** 10.3389/fmicb.2016.02054

**Published:** 2016-12-23

**Authors:** Piotr Majewski, Piotr Wieczorek, Dominika Ojdana, Anna Sieńko, Oksana Kowalczuk, Paweł Sacha, Jacek Nikliński, Elżbieta Tryniszewska

**Affiliations:** ^1^Department of Microbiological Diagnostics and Infectious Immunology, Medical University of BialystokBialystok, Poland; ^2^Department of Clinical Molecular Biology, Medical University of BialystokBialystok, Poland

**Keywords:** carbapenem-resistance, outer membrane permeability, *Enterobacter cloacae*, MLST, AmpC

## Abstract

The growing incidence of multidrug-resistant (MDR) bacteria is an emerging challenge in modern medicine. The utility of carbapenems, considered “last-line” agents in therapy of infections caused by MDR pathogens, is being diminished by the growing incidence of various resistance mechanisms. *Enterobacter cloacae* have lately begun to emerge as an important pathogen prone to exhibiting multiple drug resistance. We aimed to investigate the molecular basis of carbapenem-resistance in 44 *E. cloacae* clinical strains resistant to at least one carbapenem, and 21 susceptible strains. Molecular investigation of 65 E. cloacae clinical strains was based on quantitative polymerase chain reaction (qPCR) allowing for amplification of *ampC, ompF*, and *ompC* transcripts, and analysis of nucleotide sequences of alleles included in MLST scheme. Co-operation of three distinct carbapenem resistance mechanisms has been reported—production of OXA-48 (5%), AmpC overproduction (97.7%), and alterations in outer membrane (OM) transcriptome balance. Carbapenem-resistant *E. cloacae* were characterized by (1.) downregulation of *ompF* gene (53.4%), which encodes protein with extensive transmembrane channels, and (2.) the polarization of OM transcriptome-balance (79.1%), which was sloped toward *ompC* gene, encoding proteins recently reported to possess restrictive transmembrane channels. Subpopulations of carbapenem-susceptible strains showed relatively high degrees of sequence diversity without predominant types. ST-89 clearly dominates among carbapenem-resistant strains (88.6%) suggesting clonal spread of resistant strains. The growing prevalence of pathogens resistant to all currently available antimicrobial agents heralds the potential risk of a future “post-antibiotic era.” Great efforts need to be taken to explore the background of resistance to “last resort” antimicrobials.

## Introduction

An enormous adaptive capacity of Gram-negative multidrug-resistant (MDR) bacteria enables them to accumulate many different mechanisms of resistance to various antimicrobial agents (Nikaido, 2009; Poole, 2011). As a result occurrence of MDR pathogens considerably reduces the opportunities for an effective treatment of infectious diseases (Kaye and Pogue, 2015). Another vital epidemiological problem is the emergence and spread of novel mechanisms of antimicrobial drug resistance, especially among subpopulations of pathogens persisting in hospital environments (Hawkey and Jones, 2009; Davin-Regli and Pagès, 2015). The prevalence of those highly resistant microorganisms contributes significantly to prolonged hospitalization and increased mortality (Cerceo et al., 2016; MacVane, 2017). Increasing drug resistance among bacteria forces us to search for new therapeutic solutions and make decisions beyond standard treatment patterns (Fischbach, 2011; Tamma et al., 2012; Khameneh et al., 2016). However, the growing prevalence of pathogens resistant to most or even all currently available antimicrobial agents heralds the potential risk of a future “post-antibiotic era” (Falagas and Bliziotis, 2007; Majewski et al., 2012). According to the estimates published in a UK report on the development of antimicrobial resistance, in 2050, infections caused by MDR microorganisms could become one of the most important causes of mortality worldwide (10 million deaths per year), surpassing even the mortality rate currently caused by cancer (8.2 million deaths per year) (Review on Antimicrobial Resistance, 2014).

Carbapenems, broad spectrum agents with high bactericidal activity, are often referred to as “drugs of last resort” that retain activity against MDR Gram-negative bacteria (Papp-Wallace et al., 2011). However, the utility of carbapenems is being diminished by the growing incidence of various resistance mechanisms in bacteria (Giamarellou, 2010; Karaiskos and Giamarellou, 2014; Tängdén and Giske, 2015). Most frequently, carbapenem-resistance among *Enterobacteriaceae* is the result of various interacting β-lactam resistance strategies—namely, production of acquired carbapenemases, alteration in OM permeability, significantly increased production of chromosomally encoded β-lactamases (with slight carbapenemase activity, i.e., AmpC), and/or active efflux (Papp-Wallace et al., 2011). *E. cloacae* have lately begun to emerge as an important pathogen prone to exhibiting multiple drug resistance mechanisms and represents particularly high risk in the healthcare setting (Davin-Regli and Pagès, 2015). Therefore, we aimed to investigate the molecular basis of carbapenem-resistance in clinical strains of *E. cloacae*. Molecular characterization was based on qPCR, which was utilized in order to determine influence of chromosomal cephalosporinase (*ampC*) and porin-encoding (*ompF, ompC*) genes transcription level on carbapenem-resistance.

## Materials and methods

### Identification, susceptibility testing, and resistance detection

This study aimed to investigate the molecular basis of carbapenem resistance in 44 *E. cloacae* clinical strains resistant to at least one carbapenem, and 21 susceptible strains. Pathogens originated from patients hospitalized between 2007 and 2015 in University Hospital and the Children's University Hospital of Bialystok. Biochemical identification was performed using ID-GN cards and automated the VITEK2 system (bioMérieux, Marcy l'Etoile, France) following manufacturer's guidelines. Antimicrobial activity of carbapenems (*ertapenem, meropenem, imipenem, doripenem, biapenem*) and cephalosporins (*cefepime, cefotaxime, ceftazidime, ceftriaxone*) was investigated with the use of the microdilution method in Mueller-Hinton Broth (Oxoid, Basingstoke, UK). Results were interpreted in accordance with the European Committee on Antimicrobial Susceptibility Testing (EUCAST) (The European Committee on Antimicrobial Susceptibility Testing, 2015). Synergy testing of carbapenems (meropenem and imipenem) with β-lactamase inhibitors, double-disk synergy test for extended-spectrum β-lactamase (ESBL) screening (Mueller-Hinton with and without cloxacillin), and a biochemical carbapenemase assay (CARBA-NP test II) were performed, as described previously (Dortet et al., 2012). Polymerase chain reaction (PCR) experiments were done in order to detect various carbapenemase-encoding genes. Oligonucleotides and thermal conditions are presented in Table 1.

**Table 1:** **Oligonucleotides used in polymerase chain reactions**.

**Oligonucleotide**	**Sequence (5′–3′)**	**Product**	**Application**	**References**
OXA−48−like_F	ATC ACA GGG CGT AGT TGT GC	182 bp	PCR	This study
OXA−48−like_R	GCG TCT GTC CAT CCC ACT TA			
NMC-A/IMI_F	CAA TGG CAG GAT TGG TGT CT	412 bp	PCR	This study
NMC-A/IMI_R	CTC ATC GCC TGG AAT AGC TG			
OXA-51-like_F	GGA AGT GAA GCG TGT TGG TT	224 bp	PCR	This study
OXA-51-like_R	CCC AAC CAC TTT TTG CGT AT			
OXA-58-like_F	AAT TGG CAC GTC GTA TTG GT	231 bp	PCR	This study
OXA-58-like_R	CCC CTC TGC GCT CTA CAT AC			
GES_F	CGA AAA AGC AGC TCA GAT CG	184 bp	PCR	This study
GES_R	GTC CGG CCC ATA TGA AAG TT			
VIM_F	GAT GGT GTT TGG TCG CAT A	390 bp	PCR	Ellington et al., 2007
VIM_R	CGA ATG CGC AGC ACC AG			
KPC_F	ATG GCC GCT GGC TGG CTT TT	785 bp	PCR	Sacha et al., 2012
KPC_R	CGG CCT CGC TGT GCT TGT TCA			
IMP_F	GGA ATA GAG TGG CTT AAY TCT C	188 bp	PCR	Ellington et al., 2007
IMP_R	CCA AAC YAC TAS GTT ATC T			
NDM_F	GAC CGA TGA CCG CCC AG	372 bp	PCR	Findlay et al., 2012
NDM_R	GAC TTG GCC TTG CTG TCC TT			
*ampC_*F	CTC ACT TAA GCA GGG CAT CG	167 bp	qPCR	This study
*ampC*_R	TCA CTT CTA CCA CGG GCA AC			
*ompF*_F	GAC GCA GGC TCC TTC GAC TA	172 bp	qPCR	This study
*ompF*_R	CAA CCA GGC CGA AGA AGT TG			
*ompC*_F	CTA CGG CGT TGT TTA CGA TGT G	150 bp	qPCR	This study
*ompC*_R	AGA CCA TCA ACC AGA CCG AAG A			
*rpoB*_F	TCC ACT CAT GAC GGA CAA CG	170 bp	qPCR	This study
*rpoB*_R	GCC ATG AAC CAC GGT AAG GA			

*Detection of carbapenemase-encoding genes was carried out in the following thermal conditions—5 min of initial denaturation at 95°C, 30 cycles of denaturation at 95°C for 15 s, annealing at 55°C for 30 s, and elongation at 72°C for 30–40 s, followed by final elongation at 72°C for 5 min; Analysis of mRNA levels was carried out with the following thermal conditions—5 min of initial denaturation at 95°C, 35 cycles of denaturation at 95°C for 15 s, annealing at 55°C for 30 s, and elongation at 72°C for 30 s, followed by analysis of melting profiles of obtained products*.

### Analysis of gene expression patterns

Overnight cultures of *E. cloacae* isolates on Luria Broth (A&A Biotechnology, Gdynia, Poland) were centrifuged and subjected to total RNA isolation procedure (Total RNA Mini Plus, A&A Biotechnology, Gdynia, Poland). Traces of DNA were removed with the use of DNase and silica columns (Clean-Up RNA Concentrator, A&A Biotechnology). Quantity of total RNA extracts was examined with the use of spectrophotometer (NanoDrop™ 2000, Thermo Fisher Scientific, Waltham, USA). Synthesis of cDNA was performed with the use of 1.0 μg of total RNA, 200U of SuperScript® IV reverse transcriptase, 4 μl of concentrated Super-Script buffer (Thermo Fisher Scientific, Waltham, USA), 100 μM of deoxynucleotide triphosphates (dNTPs), 50 μM of random hexamers, 40U of RNase inhibitor and 100 μM of dithiothreitol (DTT) (A&A Biotechnology).

Real-time quantitative PCR was performed using SYBR® Green I assay with analysis of dissociation curve (Real-Time 2xPCR Master Mix SYBR C, A&A Biotechnology) on an MxPro 3005P thermal cycler (Agilent Technologies, Waldbronn, Germany). Oligonucleotides and thermal conditions are presented in Table 1. Efficiency of particular reactions were established by standard curve method. Results were calculated by efficiency corrected method described by Pfaffl (2001). Analysis of mRNA levels was carried out in triplicate. Moreover, the Liquid Handling Robot QIAgility (Qiagen, Hilden, Germany) was utilized to set up real-time quantitative PCR. *E. cloacae* ATCC 700323 (CL7094, Oxoid Culti-Loops®, Basingstoke, UK) was used as a reference in the analysis of relative changes in gene expression.

During the analysis of relative changes in gene expression level, a logarithmic transformation of fold changes (FC—fold change) was applied for statistical purposes. Quantitative (FC) and categorical data were utilized to assess differences between carbapenem-resistant and carbapenem-susceptible subpopulations. For the purpose of categorization in the analysis of relative increase of AmpC β-lactamase expression level, a threshold of log_2_FC ≥ 2.0 was adopted. For the purpose of qualitative directional analysis of relative changes in OM protein-encoding genes, three ranges of values were adopted: (1) log_2_FC ≤ −1.0 for relatively increased expression level, (2) log_2_FC ≥ 1.0 for relatively decreased expression level, and (3) log_2_FC ranging from −1.0 to 1.0 for relatively indifferent expression level.

OM transcriptome profiles of tested strains were established after the interpretation of relative changes in porin-encoding gene expression level, in accordance with accepted thresholds. OM transcriptome profiles were created in order to illustrate the relationship between the transcript level of two major porins and the phenotype. Nine possible OM transcriptome variants were adopted (Table 2).

**Table 2:** **Distribution of OM transcriptome profile among *E. cloacae* strains**.

**OM transcriptome profile**		**Polarization**	**CARB**−**R**		**CARB**−**S**	
			***n***	**[%]**	***n***	**[%]**
*ompF*(↓):*ompC*(↓)	(I)	–	1	2.3	1	4.8
*ompF*(↓):*ompC*(↑)	(II)	OmpC	11	25.6	1	4.8
*ompF*(↓):*ompC*(–)	(III)	OmpC	11	25.6	2	9.5
*ompF*(–):*ompC*(↑)	(IV)	OmpC	12	27.9	4	19
*ompF*(–):*ompC*(↓)	(V)	OmpF	3	7.0	4	19
*ompF*(–):*ompC*(–)	(VI)	–	4	9.3	6	28.6
*ompF*(↑):*ompC*(↓)	(VII)	OmpF	0	0	2	9.5
*ompF*(↑):*ompC*(↑)	(VIII)	–	1	2.3	0	0
*ompF*(↑):*ompC*(–)	(IX)	OmpF	0	0	1	4.8

*OM transcriptome profiles of tested strains were established after the interpretation of relative changes in porin-encoding gene expression level, in accordance with accepted thresholds*.

**Figure 1:**
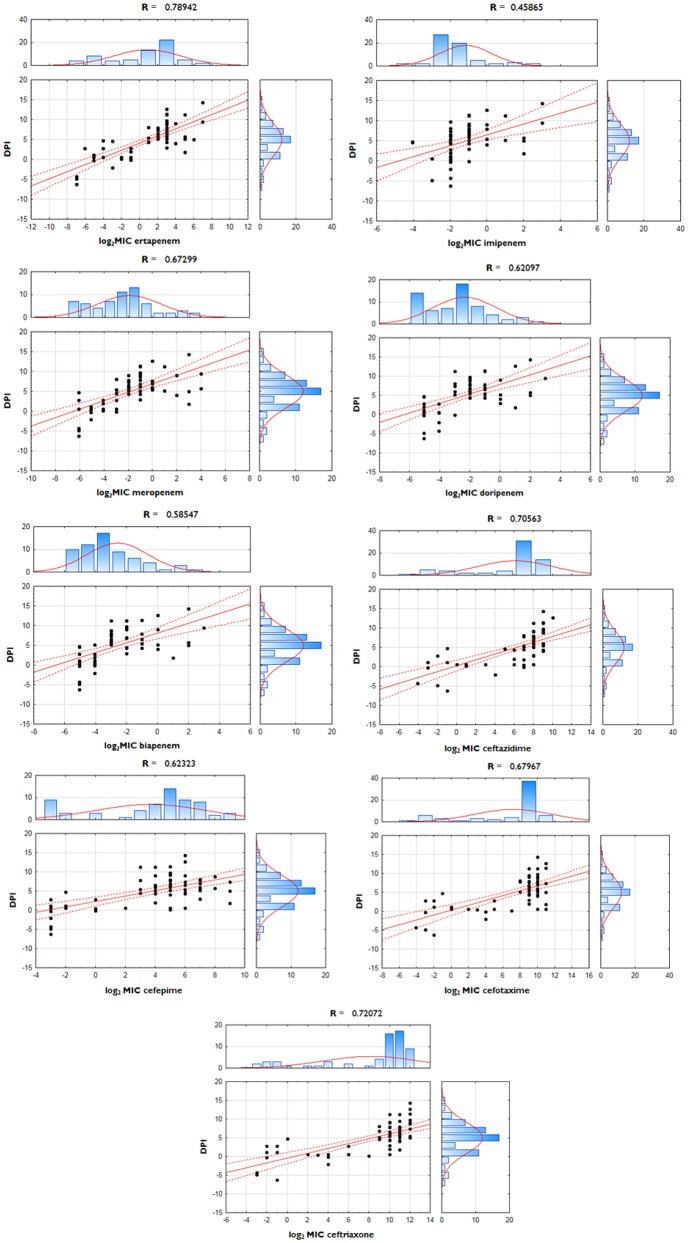
**Correlation between derepression-polarization index (DPI) and MICs among ***E. cloacae*** strains**. Correlations between log-transformed MIC values and DPI were assessed with use of Pearson correlation test.

Polarization index (PI—quotient of FC_*ompF*_ and FC_*ompC*_) was developed for the quantitative measurement of OmpC-directed OM transcriptome polarization which could have a potential link to the development of resistance to β-lactams. Polarization index differences between groups were proven to be statistically significant (Table 3, *p* = 0.0001). Moreover, the interplay between OmpC-directed OM transcriptome polarization and overexpression of AmpC β-lactamase, as well as its influence on susceptibility patterns was estimated by derivative derepression-polarization index (DPI − log_2_FC_*ampC*_ + log_2_PI). Correlation between MIC values of carbapenems and DPI values was assessed by the Pearson correlation test (Figure 1, *p* < 0.05).

**Table 3:** **Outer membrane transcriptome polarization index (PI) among *E. cloacae* strains**.

		**FC***_ompF_**/*****FC***_ompC_*			
**#**			**#**		
1	**R1**	102.6	33	**R35**	3.8
2	**R2**	2.4	34	**R36**	5.7
3	**R3**	7.7	35	**R37**	3.3
4	**R5**	0.5	36	**R38**	3.6
5	**R6**	2.7	37	**R39**	6
6	**R7**	0.4	38	**R40**	9
7	**R8**	12	39	**R41**	4
8	**R9**	5.7	40	**R42**	5.3
9	**R10**	6.3	41	**R43**	3
10	**R11**	12.5	42	**R44**	2.4
11	**R12**	11.7	43	**R69**	9.6
12	**R13**	24	44	**S45**	6.5
13	**R14**	6.5	45	**S46**	12.5
14	**R15**	8.5	46	**S47**	2.4
15	**R16**	15.5	47	**S48**	1.1
16	**R17**	20.8	48	**S49**	0.6
17	**R18**	34.1	49	**S52**	2.1
18	**R19**	2.1	50	**S53**	0.01
19	**R20**	6.5	51	**S54**	0.01
20	**R21**	1.2	52	**S55**	2.3
21	**R22**	2.8	53	**S56**	0.003
22	**R23**	1.6	54	**S57**	1.7
23	**R24**	23	55	**S58**	0.7
24	**R25**	53.7	56	**S59**	0.8
25	**R26**	1.6	57	**S61**	0.9
26	**R28**	3	58	**S62**	16.3
27	**R29**	0.5	59	**S63**	0.5
28	**R30**	1.6	60	**S64**	1.2
29	**R31**	3.6	61	**S65**	0.9
30	**R32**	0.6	62	**S66**	2.7
31	**R33**	5.5	63	**S67**	0.1
32	**R34**	10	64	**S68**	1.4

### Strain typing

Investigation of genetic relatedness was performed according to multi-locus sequence analysis scheme developed by Miyoshi-Akiyama et al. (2013). Analysis of nucleotide sequences was undertaken using Sanger sequencing (BrightDye™ Terminator Sequencing Kit, Nimagen, Nijmegen, Netherlands) and subsequent capillary electrophoresis using the 3500 Genetic Analyzer (Applied Biosystems, Foster City, USA). Any novel alleles or sequence types (STs) were assigned and stored in the PubMLST domain (http://pubmlst.org). Phylogenetic analysis with the use of concatenated sequences and maximum parsimony method (BioNumerics 7.6—evaluation license, Applied Maths, Sint-Martens-Latem, Belgium) were utilized to determine the degree of relatedness between particular strains of *E. cloacae*.

## Results

Antimicrobial susceptibility testing of 65 clinical strains of *E. cloacae* was performed with the use of the broth microdilution method (Table 4). Pathogens were divided into two groups according to their susceptibility—44 *E. cloacae* clinical strains resistant to at least one carbapenem (CARB-R), and 21 susceptible strains (CARB-S). The range of obtained MICs of particular antimicrobial agents was illustrated by MIC_50_ and MIC_90_ values. Descriptive statistics considering antimicrobial susceptibility testing are presented in Table 5.

**Table 4:** **MIC values (mg/L) of β−lactams against the clinical *E. cloacae* strains**.

	**ETP**	**IMP**	**MEM**	**DOR**	**BIA**	**CAZ**	**FEP**	**CTX**	**TRX**
**R1**	128	8	16	8	8	512	32	512	2048
**R2**	32	4	16	4	4	512	256	1024	2048
**R3**	64	4	4	4	1	8	256	512	512
**R4**	16	2	4	1	0.25	4	256	256	512
**R5**	32	4	8	2	2	128	512	2048	2048
**R6**	64	4	8	4	4	256	512	2048	4096
**R7**	32	2	2	1	1	256	32	1024	2048
**R8**	8	0.25	0.5	0.25	0.25	512	32	1024	4096
**R9**	8	0.5	1	0.25	0.125	256	128	1024	2048
**R10**	8	0.5	0.25	0.125	0.125	256	64	1024	2048
**R11**	8	0.5	0.5	0.25	0.125	256	128	1024	2048
**R12**	8	0.5	1	0.5	0.25	256	128	1024	2048
**R13**	128	8	8	4	4	512	64	1024	4096
**R14**	8	0.5	1	0.25	0.125	256	32	512	2048
**R15**	16	1	4	1	1	512	64	1024	4096
**R16**	8	0.5	0.5	0.25	0.125	512	256	1024	4096
**R17**	32	2	2	0.5	0.25	512	16	1024	2048
**R18**	8	0.5	0.5	0.5	0.125	128	8	256	1024
**R19**	4	0.25	0.25	0.125	0.06	128	16	256	512
**R20**	8	0.5	0.5	0.5	0.5	512	32	2048	4096
**R21**	16	1	4	1	1	512	32	1024	2048
**R22**	8	1	1	0.25	0.125	512	128	1024	2048
**R23**	8	0.5	0.5	1	0.25	256	128	512	1024
**R24**	8	0.5	0.5	0.25	0.25	256	32	512	1024
**R25**	8	1	1	2	1	1024	64	2048	4096
**R26**	4	0.25	0.25	0.25	0.125	128	8	512	1024
**R28**	2	0.25	0.125	0.125	0.125	256	32	512	1024
**R29**	2	0.25	0.125	0.125	0.125	128	128	256	512
**R30**	8	0.5	0.5	0.25	0.25	128	8	512	1024
**R31**	8	0.5	0.5	0.5	0.125	256	128	1024	2048
**R32**	8	0.5	0.25	0.25	0.25	64	16	512	1024
**R33**	8	0.5	0.25	0.25	0.25	256	16	512	1024
**R34**	8	0.5	0.25	0.25	0.125	256	128	512	2048
**R35**	4	0.5	0.25	0.25	0.125	256	32	512	1024
**R36**	4	0.25	0.25	0.25	0.125	512	16	512	1024
**R37**	2	0.25	0.125	0.5	0.5	256	64	512	2048
**R38**	8	0.5	0.5	0.5	0.5	512	64	1024	4096
**R39**	4	0.25	0.25	0.125	0.125	256	8	512	1024
**R40**	4	1	0.5	0.25	0.5	256	16	512	1024
**R41**	4	0.25	0.5	0.25	0.5	256	32	512	1024
**R42**	4	0.25	1	0.25	0.125	256	32	512	2048
**R43**	2	0.25	0.25	0.125	0.125	256	512	2048	4096
**R44**	8	0.5	0.5	0.5	0.5	512	64	1024	2048
**R69**	4	0.5	0.25	0.25	0.25	256	32	512	512
**S45**	0.06	0.25	0.06	0.03	0.03	0.25	0.125	0.25	0.5
**S46**	0.125	0.06	0.125	0.03	0.03	32	64	512	512
**S47**	0.5	0.25	0.06	0.03	0.06	64	16	512	1024
**S48**	0.5	0.25	0.03	0.06	0.06	128	1	8	8
**S49**	0.06	0.25	0.015	0.03	0.06	64	32	1024	1024
**S52**	0.03	0.25	0.015	0.03	0.06	8	64	2048	1024
**S53**	0.008	0.125	0.015	0.03	0.03	0.25	0.125	0.125	0.125
**S54**	0.008	0.25	0.015	0.06	0.03	0.06	0.125	0.06	0.125
**S55**	0.015	0.25	0.015	0.03	0.06	0.25	0.125	0.125	0.25
**S56**	0.008	0.25	0.015	0.03	0.03	0.5	0.125	0.25	0.5
**S57**	0.03	0.25	0.03	0.03	0.06	0.5	0.25	1	0.5
**S58**	0.25	0.25	0.06	0.06	0.06	1	0.125	1	16
**S59**	0.03	0.25	0.03	0.03	0.03	0.125	0.125	0.125	0.25
**S61**	0.25	0.25	0.06	0.03	0.03	2	32	128	256
**S62**	0.06	0.06	0.015	0.03	0.03	0.5	0.25	0.5	1
**S63**	0.5	0.5	0.125	0.125	0.06	128	1	16	16
**S64**	0.5	0.25	0.125	0.06	0.06	2	0.25	4	4
**S65**	0.03	0.125	0.03	0.03	0.03	256	4	32	64
**S66**	0.5	0.25	0.125	0.06	0.06	256	1	32	64
**S67**	0.125	0.25	0.03	0.06	0.06	16	0.125	16	16
**S68**	0.03	0.25	0.03	0.03	0.03	0.125	0.125	0.25	0.25

*ETP, ertapenem; IMP, imipenem; MEM, meropenem; DOR, doripenem; BIA, biapenem; CAZ, ceftazidime; FEP, cefepime; CTX, cefotaxime; TRX, ceftriaxone; Presented data is expressed in in mg/L*.

**Table 5:** **Descriptive statistics considering antimicrobial susceptibility of tested *Enterobacter cloacae* strains**.

**Group**	**Agent**	**Range**	**Median**	**Mode**	**MIC_50_**	**MIC_90_**
CARB−R	ETP	2–128	8	8	32	8
	IMP	0.25–8	0.5	0.5	4	0.5
	MEM	0.125–16	0.5	0.5	8	0.5
	DOR	0.125–8	0.25	0.25	4	0.25
	BIA	0.06–8	0.25	0.125	2	0.25
	CAZ	4–1024	256	256	512	256
	FEP	8–512	48	32	256	32
	CTX	256–2048	512	512	2048	512
	TRX	512–4096	2048	2048	4096	2048
CARB−S	ETP	0.008–0.5	0.06	0.03	0.5	0.06
	IMP	0.06–0.5	0.25	0.25	0.25	0.25
	MEM	0.008–0.125	0.03	0.015	0.125	0.03
	DOR	0.03–0.125	0.03	0.03	0.06	0.03
	BIA	0.03–0.06	0.045	0.06	0.06	0.06
	CAZ	0.06–256	1.5	0.25	256	2
	FEP	0.125–64	0.25	0.125	64	0.25
	CTX	0.06–2048	2.5	0.125	512	1
	TRX	0.125–1024	6	0.25	1024	4

*ETP, ertapenem; IMP, imipenem; MEM, meropenem; DOR, doripenem; BIA, biapenem; CAZ, ceftazidime; FEP, cefepime; CTX, cefotaxime; TRX, ceftriaxone; Presented data is expressed in in mg/L*.

Phenotypic double-disk synergy test revealed similar rates of ESBL occurrence in both carbapenem-resistant (41%, *n* = 18) and carbapenem-susceptible (48%, *n* = 10) subpopulations of clinical *E. cloacae* strains (Table 6). Synergy testing with the use of carbapenemase inhibitors was negative for all tested *E. cloacae* clinical strains. However, the CARBA-NP II biochemical assay revealed carbapenemase activity in two carbapenem-resistant strains (5%, *n* = 2), particularly R3 and R4. PCR and Sanger sequencing enables us to classify carbapenemase as a class D enzyme, namely OXA-48 in both carbapenemase-positive strains.

**Table 6:**
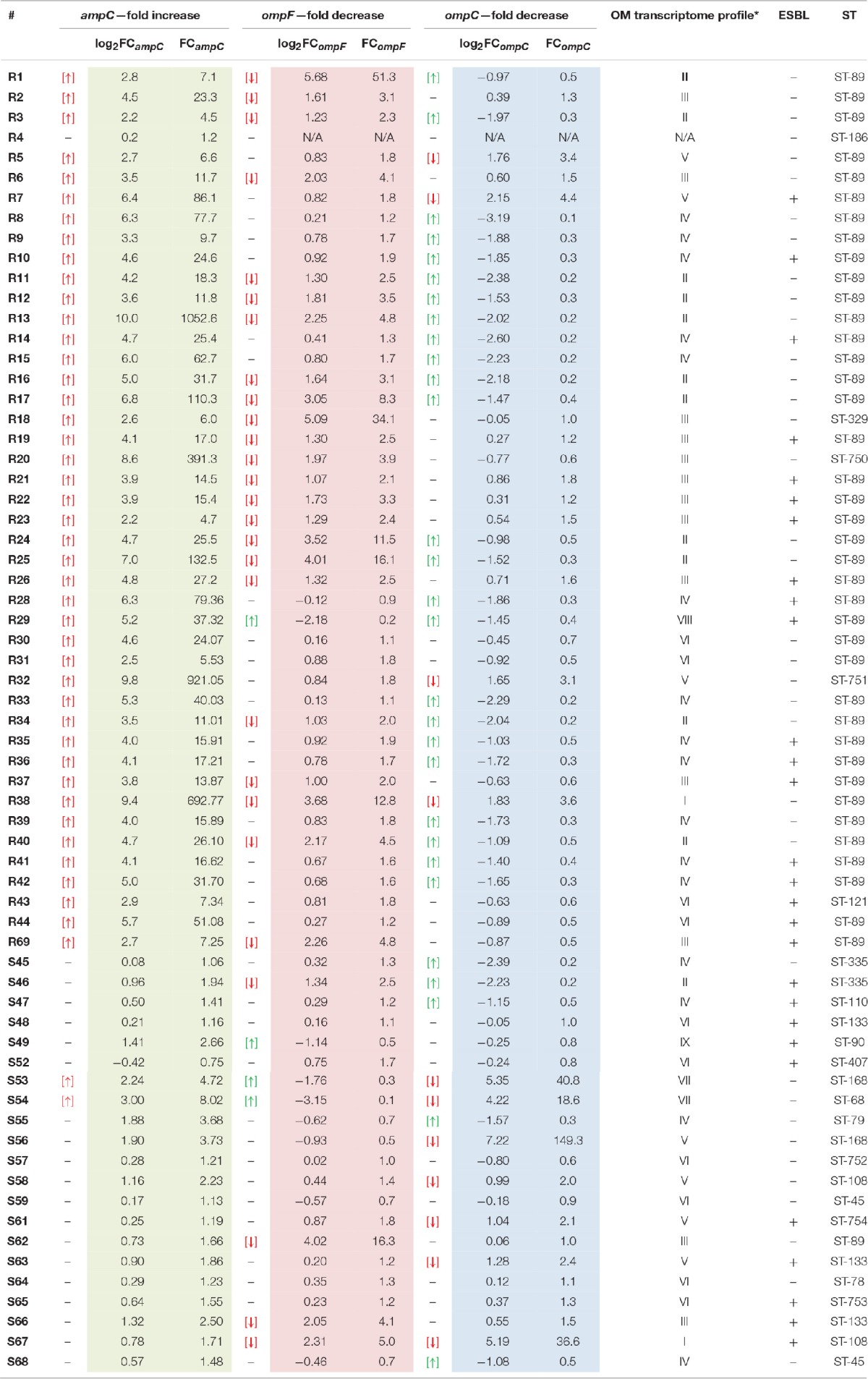
**Molecular characteristic of clinical *E. cloacae* strains**.

*Strains R3 and R4 possessed bla_OXA−48_ carbapenemase; Arrows represents up− and down−regulation of particular transcripts*.

^*^Detailed characteristics of OM transcriptome profiles is available in Table 2.

In the following step, an analysis of relative changes in the expression levels of genes encoding general OM proteins responsible for β-lactam penetration (*ompC* and *ompF*) as well as chromosomal AmpC β-lactamase was performed. Numerical data illustrating directional relative changes in gene expression levels are presented in Table 6.

Relative changes in the *bla*_*ampC*_ transcript levels in the examined *E. cloacae* subpopulations were unidirectional. Most pathogens from the CARB-R subpopulation (97.7%) showed derepression of chromosomal class C β-lactamase. Carbapenemase-producing *E. cloacae* R4 was the only nonderepressed strain accounted for in the carbapenem-resistant group. The range of log_2_FC_*ampC*_ values in the CARB-R group was between 10.0 and 0.2, with a median value at 4.35. In the CARB-S subpopulation, reported relative changes in *ampC* gene expression fell within a range of 3.0–0.4, with a median value of 0.73. Analysis of relative changes in *ampC* expression level showed statistically significant variation between CARB-R and CARB-S subpopulations (*p* < 0.000001).

During the analysis of relative changes in transcript levels (*ompF* and *ompC*) among tested *E. cloacae* strains, bidirectional changes were reported. Strain R4 was excluded from OM transcript level analysis due to lack of *ompF*, and *ompC* amplification, despite the confirmed oligonucleotide specificity on gDNA of all tested strains. Most of the CARB-R strains showed relative decrease in *ompF* expression level (53.4%). Interestingly, strains exhibiting a decrease in *ompF* expression level were present also in the CARB-S subpopulation (19%), although these pathogens did not develop simultaneous AmpC derepression. The range of log_2_FC_*ompF*_ values in the CARB-R group was between −2.18 and 5.68, with a median value at 1.03. In the CARB-S subpopulation, reported relative changes in *ompC* gene expression fell within a range of −3.15 to 4.02, with a median value of 0.23. Analysis of relative changes in *ompF* gene expression showed statistically significant differences between tested *E. cloacae* subpopulations (*p* < 0.0006). The relative decrease in *ompC* expression was present with much stronger frequency in CARB-S (33.3%), as compared to CARB-R (9%). However, most of the CARB-R strains (55.8%) were characterized by relatively increased *ompC* gene expression level. The range of log_2_FC_*ompC*_ values in the CARB-R group was between −3.19 and 2.15, with a median value at −1.03. In the CARB-S subpopulation, reported relative changes in *ompC* gene expression level fell within a range of −2.39 to 7.22, with a median value of 0.06. Analysis of relative changes in *ompC* gene expression showed statistically significant differences between tested *E. cloacae* subpopulations (*p* < 0.008).

Nine OM transcriptome profiles created in order to illustrate the relationship between the transcript level of two major porins and the phenotype are presented in Tables 5, 6. OM transcriptome profiles II, III, and IV with *ompC*-directed polarization (narrow transmembrane channel) were characteristic for the CARB-R subpopulation and constituted a total of 79.1%. In CARB-S strains OM transcriptome profiles V, VII, and IX with *ompF*-directed polarization accounted for 33.3%. Profile VI with unaltered levels of both porin-encoding genes was assigned for 28.6% of CARB-S strains. Polarization indices representing quantitative measurement of OmpC-directed OM transcriptome polarization, which could have a potential link to the development of resistance to β-lactams, are presented in Table 3 (*p* = 0.0001). Moreover, the interplay between OmpC-directed OM transcriptome polarization and overexpression of AmpC β-lactamase, as well as its influence on MIC values estimated by derivative derepression-polarization index (DPI − log_2_FC_*ampC*_ + log_2_PI). Correlations between MIC and DPI values assessed by the Pearson correlation test are presented in Figure 1 (*p* < 0.05).

**Figure 2:**
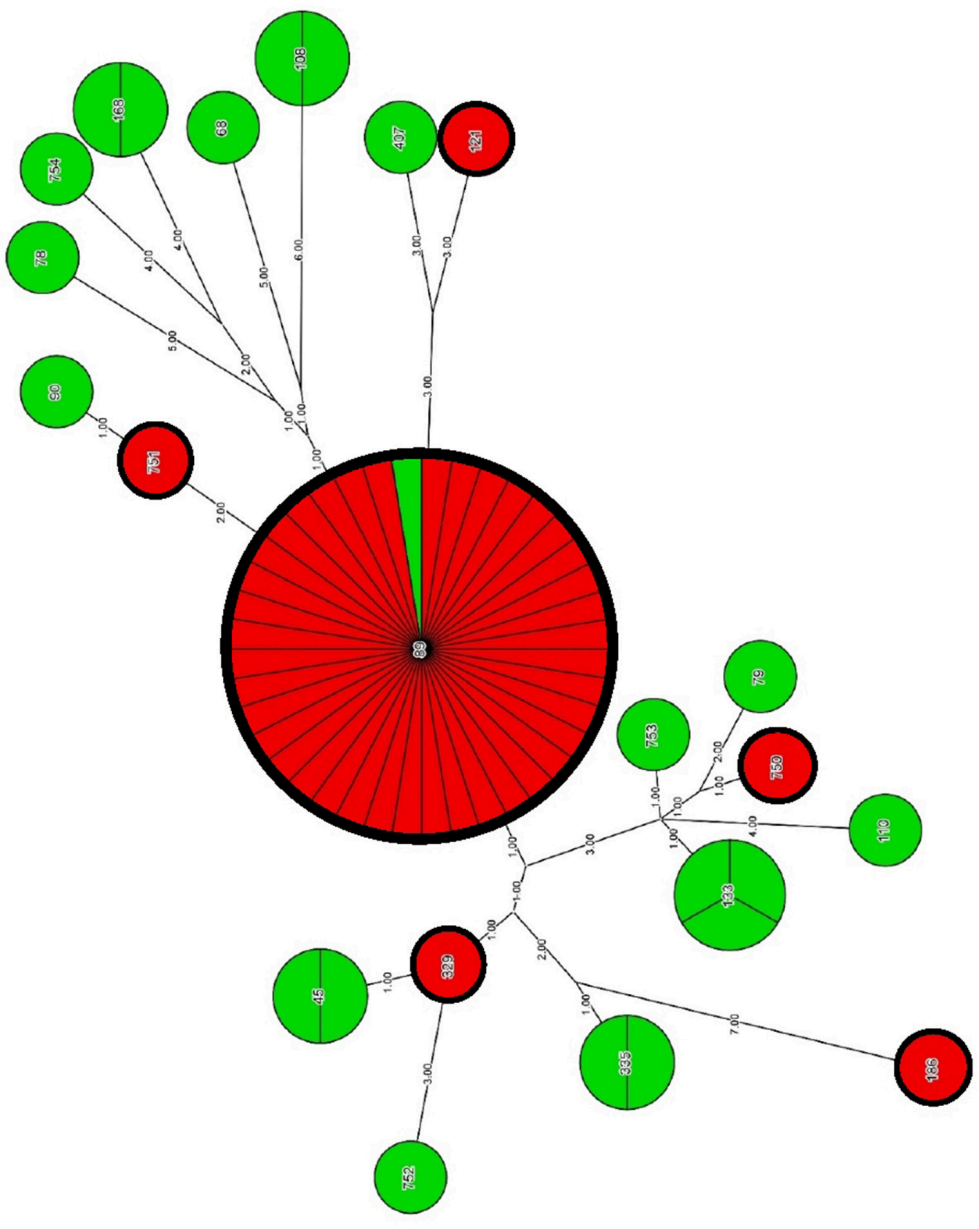
**Genetic relatedness of CARB-R and CARB-S ***E. cloacae*** strains**. Carbapenem-resistant and carbapenem-susceptible strains has been represented by red and green segments, respectively. Resolution of individual nodes in the phylogenetic tree represents the number of strains belonging to a particular ST. The number of strains attributable to a particular ST is also indicated by the division of each node into the proper amount of segments. Genetic distance between different STs is reflected by the numerical values posted at the branches of the phylogenetic tree.

Phylogenetic analysis with the use of concatenated sequences and maximum parsimony method showed a degree of relatedness between particular strains of *E. cloacae* (Figure 2, Table 6). A group of CARB-S strains was characterized by a high diversity of STs, among which we failed to specify the dominant group. Carbapenem-susceptible strains were assigned to ST-133 (14.3%), ST-335, ST-45, ST-108, ST-168 (9.5%), and single cases of ST-752, ST-753, ST-754, ST-110, ST-79, ST-407, ST-68, ST-89, and ST-90 (1.9%). Among CARB-R *E. cloacae* strains, we observed a clearly dominating ST-89 (88.6%), suggesting clonal spread of carbapenem-resistant pathogens among patients of the University Hospital and the University Children's Hospital of Bialystok. The remaining strains of CARB-R *E. cloacae* belonged to the individual, relatively closely related ST-751 and ST-329 (2.3%); more distant types ST-750 and ST-121, as well as ST-186, located far away from the entire pool of tested *E. cloacae* strains.

## Discussion

We identified three distinct mechanisms that may contribute to the phenotype of resistance to carbapenems among tested *E. cloacae* subpopulations. A unique mechanism in the performed analysis was identified in two CARB-R strains (5%). Two exceptional strains, R3 and R4, possess the ability to produce acquired OXA-48 carbapenemase. The presence of OXA-48-type carbapenemase in *E. cloacae* is not a phenomenon widely described in the literature. Strain R3 was the first case of OXA-48 carbapenemase-producing *E. cloacae* infection in Poland (Majewski et al., 2014a).

In the remaining part of the CARB-R *E. cloacae* subpopulation, we observed the coexistence of two distinct mechanisms of resistance related to β-lactams: overproduction of AmpC cephalosporinase, and alterations in the expression profile of porin-encoding genes. Constitutive overproduction of the chromosomal AmpC cephalosporinase was typical for most CARB-R strains (97.7%). The exceptional strain R4 expressed inducible AmpC, and was also capable of producing OXA-48 carbapenemase. Strains belonging to the CARB-S control group were able to produce AmpC cephalosporinase at low level (90.4%), except for two strains, S53 and S54 (*see explanation below*).

The second mechanism associated with the development of resistance to carbapenems in tested *E. cloacae* subpopulations is alterations in the OM transcriptome profile responsible for permeability of the OM. A large percentage of carbapenem-resistant strains (53.4%) were characterized by a relative decrease in the expression level of *ompF*, gene encoding an protein essential for efficient β-lactam penetration into periplasmic space (Delcour, 2009; James et al., 2009; Ziervogel and Roux, 2013). Another trend identified in the CARB-R group was the altered balance of OM transcriptome (79.1%), which was polarized toward *ompC* gene, encoding proteins recently reported to possess restrictive transmembrane channels (James et al., 2009). On the other hand, the *ompF*-directed transcriptome profile was prevalent among CARB-S strains (33.3%) suggesting that permeability was determined by a proteins with transmembrane channels favorable for β-lactam penetration. The second group of CARB-S strains was characterized by a relatively unchanged OM transcriptome profile (28.6%).

Carbapenem-susceptible *E. cloacae* strains served as an important point of reference during analysis of the coexistence of various mechanisms of resistance and their effect on the phenotype of investigated pathogens. It is worth noting that among CARB-S microorganisms we described individual cases of (1) derepression of AmpC cephalosporinase (S53, S54) as well as (2) strains demonstrating *ompC*-directed polarization of OM transcriptome. However, it must be emphasized that there was no co-occurrence of the aforementioned mechanisms. Moreover, overproduction of AmpC enzyme in S53 and S54 was compensated by *ompF*-directed OM transcriptome polarization. OM permeability of those two strains was determined by porin with a relatively large transmembrane domain, wherein the electrostatic potential and molecular configuration allows efficient penetration of β-lactam antimicrobials into periplasmic space. We described another unique strain among the CARB-S subpopulation, S62-ST89 which was sequence type dominant in group resistant to carbapenems. However, strain S62 stood out in terms of molecular characteristics—the tested strain did not develop carbapenem-resistance despite the decrease in *ompF* expression and *ompC*-directed OM transcriptome polarization. Low-level AmpC expression was the distinguishing feature of strain S62-ST89.

It seems that co-operation among different mechanisms is crucial for exhibiting carbapenem resistance in clinical aspects. Carbapenems are “poor substrates” in relation to most chromosomal and plasmidic AmpC-type isoenzymes (Mammeri et al., 2008, 2010; Jacoby, 2009). However, an increase of carbapenem MIC is often a result of decreased permeability of the OM in conjunction with a significant increase in AmpC expression level and/or mutations affecting the hydrolytic properties of chromosomal β-lactamase. Interestingly, development of clinical resistance conditioned by classical carbapenemases, in many cases also requires an additional mechanism of resistance, e.g., decreased OM permeability. The results obtained by many authors pointed to a slight increase of carbapenem MICs among strains presenting an isolated resistance mechanism—including KPC, IMP, OXA-48, and VIM-type acquired carbapenemases (Cuzon and Naas, 2008; Carrër et al., 2010; Cuzon et al., 2011; Daikos and Markogiannakis, 2011; Poirel et al., 2012). Based on low MIC values of carbapenems, evaluated pathogens were often categorized as sensitive or intermediate in relation to particular agents (Yan et al., 2001; Daikos et al., 2009; Endimiani et al., 2009; Hirsch and Tam, 2010; Kumarasamy et al., 2010; Picão et al., 2013).

Research papers analyzing the impact of permeability disturbances on carbapenem-resistance phenotype development in Gram-negative bacteria showed a great variety of possible OM transcriptome profiles. The decrease of gene expression may cover an isolated decrease of *ompC* or *ompF* porin encoding genes, as well as, both major OM proteins (Bialek et al., 2010). Expression level of genes encoding OM proteins is of key importance in case of β-lactam activity, as demonstrated in a study by Doménech-Sánchez et al. (2003) on *K. pneumoniae* strain CSUB10R, which lost both OmpF, and OmpC proteins. Complementation studies showed significant reduction of β-lactam MIC values in strains with restored OmpF protein. Complemented strain represented an 8-fold decrease in MIC values of imipenem and a 128-fold decrease of cephalosporins and meropenem MIC values. Another project emphasizing key role of OmpF protein was published by Moya-Torres et al. (2014). Authors performed molecular characterization of permeability disorders in *Serratia marcescens*, and proved that OmpF protein was the most important factor contributing to changes in MIC values of β-lactams.

The results of the research work published by Babouee Flury et al. (2016) on the development of carbapenem-resistance in *E. cloacae* proved a significant contribution of the chromosomal AmpC cephalosporinase overproduction coupled with impaired OM permeability. The authors linked permeability disorders with relative decrease of gene expression and/or mutations in the structural porin-encoding genes. Nucleotide sequence analysis of genes encoding OmpC and OmpF OM proteins showed the presence of nonsense mutations and deletions within the structural region as well as the presence of insertion sequences disrupting promoters responsible for porin expression. Carbapenem-resistant strains showed a 10–40-fold relative increase in the level of *ampC* transcripts. In this study we described a relative increase in AmpC cephalosporinase gene expression among CARB-R strains; however, the range of values was ranked from 4.5-fold increase up to expression 1000-fold higher than that of the reference strain. Analysis of relative changes in porin-encoding gene expression level carried out by Babouee Flury et al. (2016) showed a 2.5-fold decrease in *ompF* level, and a 3-fold decrease in the *ompC* expression level. The extremely low *ompC* expression described by the authors amounted to a 160-fold decrease in relation to the reference strain. In this study we described carbapenem-resistance associated with relative decrease of *ompF* expression with the extreme value of the 50-fold reduction in transcript levels. Another phenomenon described in this study is the shift in the OM porin profile. Carbapenem-resistant *E. cloacae* showed a tendency to compensatory increase of *ompC* expression.

In accordance with our results, research work published by Szabó et al. (2006) indicated the involvement of 50-fold decrease in *ompF* gene expression in carbapenem-resistant *E. cloacae*. The research project published by Pérez et al. (2007) concerning carbapenem-resistant *E. cloacae* was based on the analysis of *ompF* and *ompC* gene expression levels. The authors described decreased expression of both *ompF* (5-fold) and *ompC* (500-fold) genes in resistant strains. The decrease in expression level of both major OM proteins was also recorded in the report published by Koyano et al. (2013), Philippe et al. (2015) and Jaskulski et al. (2013).

Molecular analysis of *E. aerogenes* exhibiting high MIC of imipenem (8 mg/L) carried out by Fernández-Cuenca et al. (2006) showed two coexisting mechanisms of resistance, a decrease in expression of OM protein (40 kDa) coupled with high AmpC activity. The report prepared by Doumith et al. (2009) was based on molecular analysis of resistance to ertapenem among *Enterobacter* spp., and *Klebsiella* spp. isolated in the UK. The coexistence of enzymatic barrier (AmpC overexpression or KPC carbapenemase) in conjunction with impaired permeability of OM was found to be the main cause of ertapenem resistance in *E. cloacae*. Depending on the degree of resistance to ertapenem, the authors observed various OM protein profiles—a decrease in expression of both major porins, or isolated decrease in *ompF* expression while maintaining *ompC* gene function.

Analysis of ertapenem-resistance in *E. cloacae* isolated in Taiwan indicated the possible participation of active efflux, AmpC derepression, and impaired permeability of OM (Yang et al., 2012). Acquired carbapenemase IMP-8 was detected in 5% of resistant strains, coinciding with the results of this study. Changes in porin-encoding gene expression were observed in 43.4% of strains with various porin genotypes. The largest percentage of resistant *E. cloacae* showed impaired permeability caused by a decrease in *ompF* expression level.

Analysis of the basic requirements for the development of carbapenem resistance among *E. cloacae* strains isolated in China indicated the coexistence of derepressed chromosomal AmpC β-lactamase, a decrease in *ompF* expression, and the presence of acquired VIM-2 carbapenemase in individual strains (Lee et al., 2012). In the case of *Enterobacter* spp. isolated in Chile, among different levels of carbapenem resistance, the authors described coexistence of impaired OM permeability with AmpC overexpression (Wozniak et al., 2012). We described single strains (R30, R31, R43, and R44) presenting relatively unchanged porin profile, which were resistant to ertapenem (MIC values from 2 to 8 mg/L). Ertapenem resistance in those *E. cloacae* strains was probably associated with considerable AmpC overexpression.

In a report published by Novais et al. (2012) considering ertapenem resistance in *Enterobacteriaceae*, the authors indicated two mechanisms of OM permeability disruption, namely, nonsense mutations and/or insertion sequence (IS) incorporation into structural porin-encoding genes, and production of nonfunctional proteins effecting from mutations in the third loop region of the transmembrane channel. Reuter et al. (2013) reported a frame shift and/or stop codon presence in the *ompF* gene of resistant pathogens. Goessens et al. (2013) showed that a decreased expression of two major OM proteins may not lead to the development of carbapenem resistance. In accordance with this finding, we described the case of a single strain, S67, showing a relatively decreased expression of both *ompF* and *ompC*, which retained susceptibility to carbapenems despite coexistent ESBL production. Relatively increased ertapenem MIC (0.125 mg/L) was the only characteristic feature of strain S67.

Alterations of OM protein balance (OmpF/OmpC) in the context of β-lactam resistance development have not been widely reported in the literature. However, there are numerous reports indicating porin balance regulatory cascade as an adaptive mechanism of *Enterobacteriaceae* and other Gram-negative rods, utilized in response to adverse environmental conditions such as, high osmotic pressure or changes in the availability of oxygen (Nikaido, 2003; Ruiz et al., 2006; Vogel and Papenfort, 2006). It is possible for microorganisms to regulate the OmpF-OmpC ratio, thereby choosing porins with preferable transmembrane channel diameter, electrostatic potential, and the molecular configuration inside the channel. Regulatory cascades can also allow pathogens to take over permeability by the minor porins such as OmpY or OmpK37 (Knopp and Andersson, 2015; Bystritskaya et al., 2016). Various regulatory cascades can be launched in response to changing environmental conditions. Among possible mechanisms involved in decreasing OM permeability due to OmpC-directed OM porin balance, we can mention the increased expression of *bolA* morphogene responsible for adaptive stress response in *Enterobacteriaceae* (Freire et al., 2006), and the TolC, LamB, and Dps proteins which can significantly influence OM protein network (Yang et al., 2011). Handling of the OM protein balance according to environmental factors may also be conditioned by the two-component regulatory system OmpR-EnvZ (Chhabra et al., 2012; Shimada et al., 2015). Another mechanism influencing the relationship between produced OM proteins is a two-component system CpxA-CpxR closely associated with the activity of the AtpB protein, the β-subunit of ATP synthase localized in the plasma membrane (Lin et al., 2012). Studies conducted by Batchelor et al. (2005) based on controlled mutagenesis showed that phosphorylation of the CpxR regulator in “wild-type” strains activates histidine kinase CpxA, and leads to transient polarization of OM protein balance in response to environmental stress. Moreover, the authors proved that mutations within histidine kinase CpxA showed the possible fixation of OmpC-directed OM polarization. It can be assumed that factors disturbing the physiological regulatory cascade may be associated with consistent OM protein balance polarization toward OmpC with restrictive channel or abnormal expression of both major porins (Malickbasha et al., 2010; Yang et al., 2011; Tängdén et al., 2013).

Molecular analysis of *E. aerogenes* strains isolated during imipenem treatment revealed the potential role of *ompC*-directed OM protein polarization (Lavigne et al., 2012). Analyzed strains with permeability disturbances were characterized by resistance to ertapenem, and elevated MIC values of imipenem. Moreover, the authors described strains that lost both OmpF and OmpC proteins, and developed resistance to imipenem in the clinical aspect. Studies on the expression profile of porin-encoding genes among MDR *K. pneumoniae* performed by Hasdemir et al. (2004) showed interdependence between two major porins. For most analyzed MDR microorganisms loss of OmpF expression was accompanied with the retained production of the OmpC protein conditioning permeability of the OM.

Recently published reports indicated a high incidence of carbapenem-resistant *Enterobacteriaceae* without participation of any acquired carbapenemase. The authors indicated comorbidity of OM permeability disorders with the presence of AmpC and/or extended-spectrum β-lactamases (Hasdemir et al., 2004; Drew et al., 2013; Ammenouche et al., 2014). Acquired carbapenemase participation in the development of resistance to carbapenems among *Enterobacteriaceae* was estimated in a report published by Robert et al. (2014). The analyses included pathogens resistant to at least one carbapenem isolated primarily from urinary and respiratory tracts of patients hospitalized in 71 medical centers in France. Carbapenem-resistant *E. cloacae* strains, the subject of this paper, were also obtained in the largest proportion from the urinary and respiratory tracts. In the aforementioned report, *E. cloacae* were the predominant pathogen (58.2%), and resistance to carbapenems was not conditioned by the presence of acquired carbapenemases. Among all microorganisms subjected to molecular analysis, only 12.6% of carbapenem-resistant strains possessed acquired carbapenemases. In the case of the remaining pathogens, resistance was associated with impaired OM permeability.

Here we report the co-operation of three distinct carbapenem-resistance mechanisms—production of OXA-48 carbapenemase, AmpC overproduction, and alterations in outer membrane (OM) transcriptome balance. Carbapenem-resistant *E. cloacae* subpopulations were characterized by (1) downregulation of *ompF* gene, which encodes protein with extensive transmembrane channels, and (2) the polarization of OM transcriptome-balance, which was sloped toward *ompC* gene, encoding proteins recently reported to possess restrictive transmembrane channels. The growing prevalence of pathogens resistant to most or even all currently available antimicrobial agents heralds the potential risk of a future “post-antibiotic era” (Falagas and Bliziotis, 2007; Livermore, 2009; Hornsey et al., 2010; Chen et al., 2011; Bergen et al., 2012; Diena et al., 2012; Majewski et al., 2012, 2014b; Yahav et al., 2012; Linkevicius et al., 2013; Veleba et al., 2013; Deng et al., 2014). Great effort needs to be taken to explore the background of resistance to antimicrobials of “last-resort.”

## Author contributions

PM, PW, JN, ET substantially contributed to: conception of the submitted research paper—designing and validation of experiments (PCR and qPCR)—data acquisition and interpretation (antimicrobial susceptibility testing, Sanger sequencing, qPCR, bioinformatics)—revising the manuscript. DO, AS, OK, PS substantially contributed to:—conception of the submitted research paper—validation of designed experiments (phenotypic methods)—data acquisition and interpretation (antimicrobial susceptibility testing, nucleic acid purification, PCR, bioinformatics)—drafting the manuscript.

## Funding

This study was conducted with the use of equipment purchased by Medical University of Bialystok as part of the OP DEP 2007-2013, Priority Axis I.3, contract No POPW.01.03.00-20-022/09. The paper submitted was supported in part by departmental and The Leading National Research Centre sources.

### Conflict of interest statement

The authors declare that the research was conducted in the absence of any commercial or financial relationships that could be construed as a potential conflict of interest.
